# An Inflamed Human Alveolar Model for Testing the Efficiency of Anti-inflammatory Drugs *in vitro*

**DOI:** 10.3389/fbioe.2020.00987

**Published:** 2020-08-21

**Authors:** Barbara Drasler, Bedia Begum Karakocak, Esma Bahar Tankus, Hana Barosova, Jun Abe, Mauro Sousa de Almeida, Alke Petri-Fink, Barbara Rothen-Rutishauser

**Affiliations:** ^1^Institut Adolphe Merkle, Faculté des Sciences et de Médecine, Université de Fribourg, Fribourg, Switzerland; ^2^Department of Oncology, Microbiology and Immunology, Faculty of Science and Medicine, University of Fribourg, Fribourg, Switzerland; ^3^Département de Chimie, Faculté des Sciences et de Médecine, Université de Fribourg, Fribourg, Switzerland

**Keywords:** inflammation, lung, *in vitro*, multicellular models, macrophage phenotype, anti-inflammatory drugs, corticosteroids

## Abstract

A large number of prevalent lung diseases is associated with tissue inflammation. Clinically, corticosteroid therapies are applied systemically or via inhalation for the treatment of lung inflammation, and a number of novel therapies are being developed that require preclinical testing. In alveoli, macrophages and dendritic cells play a key role in initiating and diminishing pro-inflammatory reactions and, in particular, macrophage plasticity (M1 and M2 phenotypes shifts) has been reported to play a significant role in these reactions. Thus far, no studies with *in vitro* lung epithelial models have tested the comparison between systemic and direct pulmonary drug delivery. Therefore, the aim of this study was to develop an inflamed human alveolar epithelium model and to test the resolution of LPS-induced inflammation *in vitro* with a corticosteroid, methylprednisolone (MP). A specific focus of the study was the macrophage phenotype shifts in response to these stimuli. First, human monocyte-derived macrophages were examined for phenotype shifts upon exposure to lipopolysaccharide (LPS), followed by treatment with MP. A multicellular human alveolar model, composed of macrophages, dendritic cells, and epithelial cells, was then employed for the development of inflamed models. The models were used to test the anti-inflammatory potency of MP by monitoring the secretion of pro-inflammatory mediators (interleukin [IL]-8, tumor necrosis factor-α [TNF-α], and IL-1β) through four different approaches, mimicking clinical scenarios of inflammation and treatment. In macrophage monocultures, LPS stimulation shifted the phenotype towards M1, as demonstrated by increased release of IL-8 and TNF-α and altered expression of phenotype-associated surface markers (CD86, CD206). MP treatment of inflamed macrophages reversed the phenotype towards M2. In multicellular models, increased pro-inflammatory reactions after LPS exposure were observed, as demonstrated by protein secretion and gene expression measurements. In all scenarios, among the tested mediators the most pronounced anti-inflammatory effect of MP was observed for IL-8. Our findings demonstrate that our inflamed multicellular human lung model is a promising tool for the evaluation of anti-inflammatory potency of drug candidates *in vitro*. With the presented setup, our model allows a meaningful comparison of the systemic vs. inhalation administration routes for the evaluation of the efficacy of a drug *in vitro*.

## Introduction

Lung inflammation plays an important role in the pathogenesis of a number of respiratory diseases, such as pneumonia, acute respiratory distress syndrome, or chronic inflammatory disorders such as asthma and chronic obstructive pulmonary disease (Johnson and Matthay, 2010; Roth, 2014). As the lung is the vital organ for gas exchange, excessive inflammation in the lung tissue can be life-threatening (Moldoveanu et al., 2009). Lung tissue inflammation, as well as the anti-inflammatory reactions, involve complex interactions between and among the immune cells and the structural lung cells (Nicod, 2005; Holt et al., 2008). The airways and the lung parenchyma contain dense networks of immune cells, e.g., dendritic cells and alveolar macrophages, which play a key role in regulating the body’s immune responses (Holt et al., 2008). Dendritic cells are professional antigen-presenting cells (APCs) linking innate and adaptive immunity (Banchereau and Steinman, 1998; Cook and MacDonald, 2016). Another type of APCs, macrophages, are located in the apical part of the epithelium, and their main role is professional phagocytosis (Brain, 1988; Lehnert, 1992; Hussell and Bell, 2014). Macrophages and dendritic cells are capable of triggering rapid pro-inflammatory reactions in response to inhaled foreign materials, as well as bacterial, viral, and fungal infections through the release of pro-inflammatory mediators, such as tumor necrosis factor α (TNF-α), interleukin (IL)-1β, and IL-8, along with the increased release of reactive oxygen and nitrogen species (Condon et al., 2011; Laskin et al., 2011). In particular, macrophages also play a pivotal role in the resolution of alveolar inflammation (Frankenberger et al., 2005; Moldoveanu et al., 2009; Tu et al., 2017; Lu et al., 2018). The biological activity of macrophages is mediated by their phenotypically distinct subpopulations, i.e., the M1 and M2 phenotypes, which develop and shift in response to the mediators in their microenvironment. According to the basic dichotomic macrophage polarization classification, M1 macrophage polarization, the pro-inflammatory phenotype is stimulated by specific pathogens (Atri et al., 2018). The M1 phenotype is associated with the release of pro-inflammatory mediators, with the expression of toll-like receptors (TLR-2 and TLR-4) and co-stimulatory molecules, such as cluster of differentiation (CD) 86 (Martinez and Gordon, 2014). The activity of M1 macrophages is balanced by those with M2 phenotypes through the secretion of anti-inflammatory mediators (Martinez and Gordon, 2014). The M2 subset is stimulated with anti-inflammatory agents, such as steroids (Laskin et al., 2011; Wang et al., 2014; Jaroch et al., 2018; Desgeorges et al., 2019). The M2 phenotype is associated with expression of the mannose receptor-1 (CD206) as well as macrophage scavenger receptors (CD204 and CD163) (Gordon, 2003).

Due to the distinctive roles of macrophages in the lung, they have been proposed as the main cellular targets in the treatment of lung-inflammation-related disorders (Laskin et al., 2011). Clinically, immunosuppressant drugs, such as corticosteroids, among them methylprednisolone (MP), play an integral role in anti-inflammatory treatments of pulmonary diseases, particularly in asthma, acute lung injury, and acute respiratory distress syndrome (Silva et al., 2009; Barnes, 2011; Marik et al., 2011; Liu et al., 2013). The efficacy of corticosteroids in suppressing lung inflammation has been confirmed by both systemic administration and inhalation (Sethi and Singhal, 2008; Higham et al., 2015). MP has been shown to attenuate acute lung injury, induced with bacterial endotoxin lipopolysaccharide (LPS), via promoting macrophage polarization into the M2 subset (Tu et al., 2017). Although corticosteroids are widely used agents for the treatment of lung inflammation, strong systemic side effects such as respiratory tract infections, allergies, wound healing impairment, and bronchitis have been reported (Liu et al., 2013; Waljee et al., 2017). As a result, alternative anti-inflammatory therapies are currently being investigated (da Silva et al., 2017).

The role of macrophage phenotypes in lung inflammation and its resolution has been investigated extensively in murine models *in vivo* (Leite-Junior et al., 2008; Katsura et al., 2015; Florentin et al., 2018; Lu et al., 2018). The anti-inflammatory activity of corticosteroids in murine lung epithelia has also been investigated *in vivo*, both for the treatment of allergic asthma (Klaßen et al., 2017) and in order to determine their contribution to fetal lung maturation (Habermehl et al., 2011). On the other hand, based on the findings of clinical studies, it has been proposed that distinctive macrophage phenotype populations play an important role in anti-inflammatory treatment, e.g., in that of chronic obstructive pulmonary disease with poor response to corticosteroids (Barnes, 2011; Chana et al., 2014). Nevertheless, it should be noted that dendritic cells and the alveolar epithelium also contribute significantly to glucocorticoid-mediated immunosuppressive effects (Zach et al., 1993; Piemonti et al., 1999; Rozkova et al., 2006; Klaßen et al., 2017).

With respect to human lung models, mono- and multicellular models, primary or cell line-derived, have been developed to mimic human airway epithelia and the alveolar epithelial-endothelial tissue barrier (Hermanns et al., 2004; Steimer et al., 2005; Bur and Lehr, 2008; Haghi et al., 2015; Hittinger et al., 2016; Yonker et al., 2017; Costa et al., 2019; Artzy-Schnirman et al., 2020). These models have been employed in investigations of lung inflammation to study various lung diseases and disorders, such as cystic fibrosis (Castellani et al., 2018), chronic obstructive pulmonary disorder (Haghi et al., 2015; Zhou et al., 2019), and acute respiratory distress syndrome (Viola et al., 2019). Also, these models have been employed to investigate the safety and efficacy of aerosolized and dry powder formulations for pulmonary drug delivery (Hittinger et al., 2016), including that of steroid drugs (Bur and Lehr, 2008; Haghi et al., 2015). Finally, an *in vitro* human airway model differentiated from primary human bronchial cells has been proposed for pharmacokinetics studies (Rivera-Burgos et al., 2016). LPS is a major component of the outer membrane of Gram-negative bacteria, such as *Haemophilus influenzae*, which are considered one of the main causes of infectious lung diseases (King, 2012). For induction of inflammation, LPS is often used as a stimulus to investigate lung inflammation *in vitro*, to study the efficacy of drugs or vaccinations, or to elucidate the mechanisms of the pulmonary response to bacterial ligands (Knapp, 2009; Bisig et al., 2019; Nova et al., 2019). To date, however, there have been no studies demonstrating a comparison of drug delivery approaches, i.e., systemic vs. direct pulmonary delivery of anti-inflammatory drugs, using 3D multicellular systems of human lung epithelial tissue *in vitro*.

Given this paucity and the high potential utility of such a comparative study, this study was aimed at the development of an inflamed, immunocompetent, multicellular human lung model, including macrophages and dendritic cells. The simultaneous presence of both immune cell types is important in the cellular interplay in response to exposure to an inflammatory mediator, such as the endotoxin LPS (Bisig et al., 2019; Nova et al., 2019). As a model cell type for human alveolar pneumocytes, the epithelial A549 cell line, isolated from a pulmonary adenocarcinoma, was used. A549 cells possess characteristics of squamous type II epithelial cells of the alveolar region; these cells carry lamellar bodies containing densely packed phospholipids, the constituents of the pulmonary surfactant (Shapiro et al., 1978), and release the surfactant upon exposure to air-liquid interface (ALI) (Blank et al., 2006). In this study, a widely used anti-inflammatory drug, the corticosteroid MP, was initially tested for its ability to induce macrophage phenotype shifts in primary human monocyte-derived macrophages (MDMs) from the pro-inflammatory (M1) towards the anti-inflammatory (M2) subset, and vice-versa. Then, a 3D multicellular human lung epithelial tissue barrier model composed of the same macrophage type along with an alveolar epithelial cell layer, and monocyte-derived dendritic cells (MDDCs) (Rothen-Rutishauser et al., 2005), was stimulated with LPS from either the basal or apical side, mimicking systemic or alveolar lumen-derived inflammation, respectively. These inflamed lung models were further treated with MP in the co-presence of the pro-inflammatory stimulus (LPS) applied from the opposite side of the human lung model, simulating either intravenous drug administration or aerosolized drug administration via inhalation. In the last step, the LPS was removed prior to the anti-inflammatory treatment, mimicking a lower degree of inflammation in the tissue, and the steroid therapy was applied via the respiratory route, i.e., onto the apical side of the tissue. We employed the adverse outcome pathway (AOP) concept for pulmonary fibrosis as it involves a strong inflammatory component. As a result, the focus herein is on so-called key event two (KE2), which is the release of pro-inflammatory cytokines (e.g., tumor necrosis factor-alpha [TNF-α], interleukin [IL]-1β, and IL-8) and the loss of alveolar barrier integrity (Labib et al., 2016; Vietti et al., 2016; Villeneuve et al., 2018).

Herein, we provide experimental evidence for suppression of pro-inflammatory reactions *in vitro* using a widely used anti-inflammatory drug, a corticosteroid MP. We discuss these reactions with respect to the macrophage phenotype shifts towards pro- and anti-inflammatory subsets. We demonstrate the responsiveness of our 3D human lung model to pro- and anti-inflammatory agents, both at the pro-inflammatory protein secretion and gene expression levels. The presented model may serve as a diseased human lung model for *in vitro* pre-clinical testing of the safety, potency, and efficacy of newly developed anti-inflammatory formulations and also can serve as a diseased model to assess occupational exposures to all types of aerosols.

## Materials and Methods

### Chemicals, Reagents, and Laboratory Conditions

All the chemical reagents were purchased from Sigma-Aldrich (Switzerland), while all cell culture reagents were purchased from Gibco and Thermo Fisher Scientific (Switzerland) unless stated otherwise. MilliQ water (ultrapure water of 18.2 MΩ.cm) was used in all the experiments. Methylprednisolone (MP; Cayman Chemical, Ann Arbor, MI, United States) powder was dissolved in absolute ethanol to 5 mg/mL. At every step during cell culture and exposure, cells were kept in an incubator under controlled conditions (humidified atmosphere, 37°C, 5% CO_2_). Cell numbers were determined using an EVE^TM^ bench-top automated cell counter (Witec AG, Switzerland) with the trypan blue exclusion method (0.4% trypan blue solution in phosphate buffer saline [PBS]).

### Isolation and Differentiation of Primary Human Peripheral Blood-Derived Immune Cells

Human MDMs and MDDCs were prepared from monocytes isolated from buffy coats provided by the Swiss Transfusion Center (Bern, Switzerland), as described previously (Barosova et al., 2020). In brief, isolated blood monocytes were cultured in 6-well tissue culture plates (Corning, FALCON^®^, United States) for six days at a density of 10^6^ cells/mL in 3 mL Roswell Park Memorial Institute (RPMI)-1640 cell culture medium supplemented with 10% (v/v) fetal bovine serum (42G1189K), 2 mM L-glutamine, and penicillin-streptomycin (100 units/mL and 100 μg/mL, respectively), referred to as the complete cell culture medium (cRPMI). For MDM differentiation, 10 ng/mL of macrophage colony-stimulating factor (M-CSF) was added to cRPMI. MDDC differentiation was performed in the presence of 10 ng/mL of recombinant human interleukin 4 (IL-4) and 10 ng/mL of granulocyte-macrophage colony-stimulating factor (GM-CSF; all the factors were obtained from Milteny Biotec, Germany). Experiments involving primary monocyte isolation from human blood were approved by the committee of the Federal Office for Public Health Switzerland (reference number: 611-1, Meldung A110635/2) for the Adolphe Merkle Institute.

### Macrophage Phenotype Shifts: Monoculture Experiments

#### Macrophage Challenge With Pro- and Anti-inflammatory Agents

After six days of MDMs differentiation, cRPMI containing the growth factor was aspirated and replaced with either 2.5 mL fresh cRPMI or cRPMI containing lipopolysaccharide (LPS; 1 μg/mL; isolated from *E. coli*, strain O55:B5, Sigma-Aldrich, Switzerland). After 24 h, cRPMI containing MP or vehicle was added to the cells (the summary of the treatments is presented in Table 1).

**TABLE 1:** Treatments of the differentiated MDMs.

Sample Description	0 to 24 h	24 to 48 h
Untreated cells	cRPMI (3 mL)	cRPMI not changed
Negative control	cRPMI (2.5 mL)	Vehicle (0.5 mL)
LPS	cRPMI (2.5 mL) with LPS (1 μg/mL)	Vehicle (0.5 mL)
LPS + MP10	cRPMI (2.5 mL) with LPS (1 μg/mL)	MP (0.5 mL of 0.06 mM stock in cRPMI). Final concentration 0.01 mM
MP control	cRPMI (2.5 mL)	MP (0.5 mL of 0.06 mM stock in cRPMI). Final concentration 0.01 mM
IL-4+IL-13 (M2 control)	cRPMI with IL-4 and IL-13 (20 ng/mL)	Treatment not changed
Triton X-100 (positive control for LDH)	cRPMI (3 mL)	Triton X-100 (0.3 mL). Final concentration 0.2% (v/v)

*Vehicle is a solution of absolute ethanol (0.005% in cRPMI; v/v).*

#### Flow Cytometry

##### Immunostaining for cell surface protein markers

After stimulation of MDMs, supernatant and non-adherent cells were removed, centrifuged (500 RCF, 5 min), and kept at 4°C or −80°C until being subjected to a cell viability assay or enzyme-linked immunosorbent assay (ELISA), respectively. Adherent cells were gently washed with PBS and detached from the wells using a cell scraper (SARSTEDT, United States) in fresh PBS. Cells from two identically treated wells in a 6-well plate were pooled in flow cytometry tubes (FALCON^®^ 5 mL Polystyrene Round-Bottom Tube, Corning, Switzerland), counted, centrifuged (5702R, Eppendorf; 500 RCF, 5 min), and resuspended in cold flow cytometry buffer (PBS with 1% BSA, 0.1% NaN3, 1 mM ethylenediaminetetraacetic acid [EDTA] at pH 7.4). Before antibody labeling, cells were incubated with Fc-receptor-blocking reagent (Miltenyi Biotec, Germany) according to the supplier’s protocol. After incubation for 10 min, the suspensions were split into two separate flow cytometry tubes at a density of 1 × 10^6^ cells/mL for staining or as unstained controls.

Cells were stained with Alexa Fluor 488 conjugated anti-human CD86 (B7-2) monoclonal antibody (clone IT2.2; at 1.25 μg/mL), and APC conjugated anti-human CD206 (MMR) monoclonal antibody (clone 19.2; at 3 μg/mL), as M1 and M2 macrophage markers, respectively, in cold flow cytometry buffer containing 2 μM 4,6-diamidino-2-phenylindole (DAPI; Sigma Aldrich, Switzerland) for dead cell exclusion. Both antibodies were obtained from eBioscience^TM^ (Thermo Fisher Scientific, Switzerland). Additional untreated samples were prepared for fluorescence minus one control staining using OneComp^TM^ ebeads compensation beads (Thermo Fisher Scientific, Switzerland) to set up the cytometer. After antibody labeling for 30 min at 4°C in the dark, the cells were washed with flow cytometry buffer (3 mL) and centrifuged (500 RCF, 5 min, 4°C). The cell pellet was resuspended with cold flow cytometry buffer and stored at 4°C until data acquisition (up to 2 h). Data were acquired using LSRFortessa (BD Biosciences, Switzerland) and analyzed using FlowJo software (Version 10.6.1, TreeStar, United States).

#### Cytokine Secretion

The amount of released pro-inflammatory mediators (IL-8, IL-1β, and TNF-α) was quantified using a DuoSet ELISA Development Kit (R&D Systems, Switzerland) according to the supplier’s protocol. For IL-8, all the samples were diluted 1:4 (v/v) in reagent diluent for the measured values to remain within the detection limit of the instrument. TNF-α and IL-1β were measured in polystyrene high-binding surface 96-well plates (Corning^®^, Switzerland) without dilution. Standards and samples were run in triplicate. The concentrations of the cytokines released in the cell culture medium were calculated based on the standard curves and fitted with a four-parameter logistic (4PL) approach using GraphPad Prism 8 software (GraphPad Software Inc., San Diego, CA, United States).

#### Cell Viability/Membrane Rupture

Cell viability was evaluated by the lactate dehydrogenase (LDH; cytosolic enzyme) released in cRPMI, analyzed in triplicate using LDH cytotoxicity detection kit (Roche Applied Science, Mannheim, Germany) according to the manufacturer’s protocol. The absorbance of the colorimetric product was determined spectrophotometrically (Benchmark Microplate reader, BioRad, Switzerland) at 490 nm with a reference wavelength of 630 nm with 30-s intervals for 10 measurements. The values were expressed as fold increase of slopes (0–5 min reaction) of the treated samples relative to those of untreated samples of the multicellular models.

## Multicellular Human Lung Model Assembly

The multicellular models of human alveolar epithelial type II cell line A549, MDMs, and MDDCs were prepared as previously described (Barosova et al., 2020). In brief, A549 cells were maintained in cRPMI (passage number 5 to 25). A549 cells were seeded on transparent cell culture inserts (surface area of 4.2 cm^2^, 3.0 μm pore diameter, high pore density, polyethylene terephthalate [PET] membranes for 6-well plates; Falcon, BD Biosciences, Switzerland) at a cell density of 28.0 × 10^4^ cells/cm^2^ in 2 mL of cRPMI at 58 × 10^4^ cells/mL. The membrane inserts with cells were placed in tissue culture plates (6-well plates; Falcon, BD Biosciences, Switzerland) containing 3 mL of cRPMI and cultured for four days to form a confluent monolayer. For the multicellular model composition, membrane inserts with A549 cells were removed from the 6-well plates and placed in a sterile glass petri dish turned upside down. Cells that had grown through the pores on the basal side of the inserts were gently abraded with a cell scraper. MDDCs were pipetted onto the bottom side of the inserts: 300 μL of cell suspension in cRPMI at 98.0 × 10^4^ cells/mL (29.4 × 10^4^ cells/insert, corresponding to a seeded cell density of 7 × 10^4^ cells/cm^2^) and incubated for 70 min at 37°C and 5% CO_2_. Membrane inserts were placed back into 6-well plates containing 3 mL of pre-heated fresh cRPMI. Then, 2 mL of a MDM suspension at concentration 2.95 × 10^4^ cells/mL in cRPMI was gently added on the top of the A549 cells on inserts (5.9 × 10^4^ cells/insert, corresponding to 1.4 × 10^4^ cells/cm^2^). The assembled multicellular models were exposed for 24 h to air at the ALI to allow for surfactant production by the A549 cells. MDDCs and MDMs prepared from one buffy coat donor and A549 cells from an individual passage were used for each independent experiment. The A549 cell line was purchased from the American Tissue Type Culture Collection (ATCC^®^CCL-185^TM^) and was authenticated using short tandem repeat (STR) profiling. Cells were regularly tested for the absence of mycoplasma.

### Inflamed Model: Induction and Assessment of Pro-inflammatory Reactions

The assembled multicellular model was challenged with 1 μg/mL LPS for 48 h, from either apical or basal compartments. LPS stock (1 mg/mL in water) was diluted in cRPMI. Old cRPMI was aspirated from the bottom compartments in all samples. Then, either cRPMI (3 mL) with LPS was applied to the basal compartment, and nothing to the apical (approach 1), or fresh cRPMI was added to the basal compartment and 0.2 mL of cRPMI with LPS to the apical side, referred to as pseudo-ALI conditions (Endes et al., 2014) (approach 2).

#### Inflammatory Reactions

##### Secretion of pro-inflammatory mediators

After the LPS challenge, the concentration of IL-8, IL-1β, and TNF-α released in the cell culture medium in the basal compartment was quantified by ELISA, as described for the monoculture experiments.

#### Response at Gene Expression Levels

Upon collection of supernatants in the basal compartment, models were washed with PBS, and inserts with cells were cut out of the plastic holders. Half of each insert was incubated in RNA protection buffer (Qiagen, Germany). The cells were removed from the membranes by extensive vortexing and kept at 4°C for up to seven days (the other half was fixed for immunofluorescence staining). Then, RNA isolation was performed using ReliaPrep^TM^ RNA Miniprep Systems (Promega, Switzerland) according to the manufacturer’s protocol. RNA concentrations in samples were analyzed with a NanoDrop 2000 spectrophotometer (Thermo Scientific, United States) and stored at −20°C. Complementary DNA (cDNA) was synthesized with the Omniscript RT system (Qiagen, Germany), Oligo dT (Microsynth, Switzerland), and RNasin Inhibitor (Promega, Switzerland). Reverse transcriptase reactions were performed in 10 μL volumes with an RNA concentration of 25 ng/μL (Omniscript RT, Qiagen) and Oligo dT primers (Qiagen). RNase inhibitor (0.25 μL; RNasin Plus RNase Inhibitor, Promega) was added to the reverse transcriptase reactions. A total of 2 μL of the tenfold diluted cDNA was used for real-time PCR in reaction volumes of 10 μL with SYBR Green as reporter dye (Fast SYBR Green master mix, 7500 fast real-time PCR system, Applied Biosystems, United States). The mRNA levels were calculated using the double delta of cycle threshold (ΔΔCt) method, calculated based on the expression of a standard gene (glyceraldehyde-3-phosphate dehydrogenase; GAPDH) and the respective gene expressions in untreated cells. Primer sequences and database accession numbers are listed in the Supplementary Material (Supplementary Table S1).

### Suppression of LPS-Induced Pro-inflammatory Reactions

To prepare inflamed cell-culture models, LPS was added either from the basal compartment or from the apical compartments and left to stand for 24 h, following the procedure described above. After the first 24 h, MP was added and left to stand for an additional 24 h while LPS was still present. In approach 1, the MP stock (13 mM in absolute ethanol) was diluted in PBS to the final concentrations of 10 and 100 μM, and 200 μL was applied directly to the apical side of the models. In approach 2, MP stock was diluted in the existing cRPMI in the basolateral compartment to the same final concentrations and left to stand for 24 h. For negative control samples, the vehicle, absolute ethanol, was added at a final concentration of 0.07% or 0.7% to gauge potential biological effects of the solvent alone for comparison with entirely untreated samples. LPS-treated samples were considered as the positive controls for the pro-inflammatory scenario. In parallel, the effects of MP treatments on non-inflamed models were tested following the same procedure as described in approaches 1 and 2, but without LPS in cRPMI. After treatment, the biological responses of the models were assessed by ELISA, real-time RT-qPCR, and confocal microscopy.

### Suppression of Pro-inflammatory Reactions Upon LPS Removal

**FIGURE 1:**
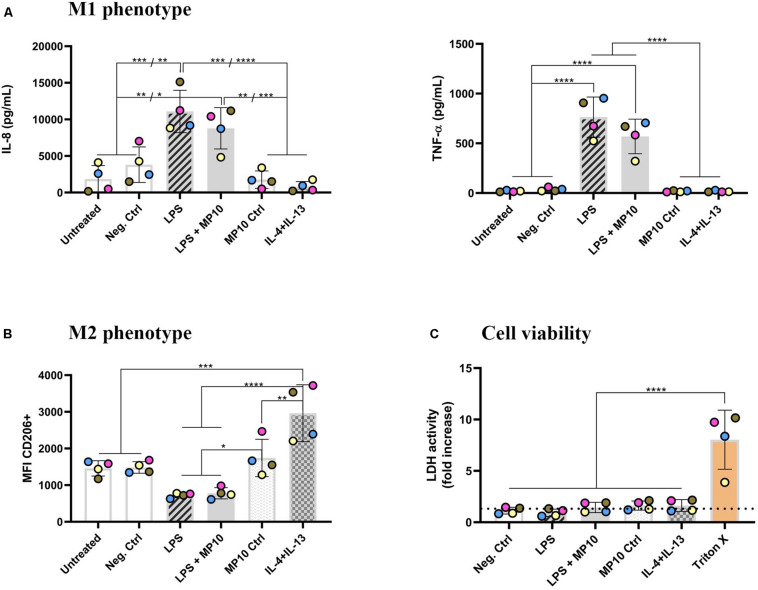
Phenotype shift in macrophage monocultures upon exposure to pro- and anti-inflammatory stimuli. **(A)** Secretion of pro-inflammatory mediators (IL-8 and TNF-α; in pg/mL) in cell-culture medium in the basal compartment, assessed via ELISA. **(B)** Expression of CD206 surface marker (median fluorescence intensity), denoting M2 phenotype, assessed via flow cytometry analysis. The CD206 intensity histogram is shown in Supplementary Figure S2. **(C)** Cell viability of MDMs after all of the treatments, assessed via membrane rupture (LDH assay), presented as fold increase over untreated cells (the dotted line). The data is presented as the mean of the four biological repetitions ± standard deviation, whereas individual values from biological repetitions are presented as circles, color-coded for the data from the same biological repetitions (donors). Statistically significant differences among the groups (One-way ANOVA, Tukey’s *post hoc*; α = 0.05): *p ≤ 0.05; **p ≤ 0.01; ***p ≤ 0.001, ****p ≤ 0.0001. Abbreviations: IL-8, interleukin 8; TNF-α, tumor necrosis factor α; MFI, median fluorescence intensity; CD206, cluster of differentiation 206, mannose receptor; IL-4+IL-13, interleukins 4 and 13 (both applied at 20 ng/mL for 48 h); LDH, lactate dehydrogenase; Pos. ctrl Triton X, positive control for LDH assay, i.e., cells exposed to 0.2% Triton X-100 (v/v; 24 h).

In approaches 3 and 4, inflamed models were established by a 24-h pre-challenge with LPS from the apical side at the pseudo-ALI (Figure 1). After 24 h, the apical sides were gently rinsed with PBS, and MP was added to the apical side at the pseudo-ALI for an additional 24 h. In approach 3, the basal cell culture medium was not changed before the MP treatment. In approach 4, the models were washed with PBS from both sides, and cRPMI in the basal compartment was refreshed. Positive controls (“LPS”) and “MP only” controls were treated with the vehicle at the pseudo-ALI. Upon completion of the treatment, the biological response was assessed as described above.

### Multicellular Model Visualization

Tissue morphology of both LPS-challenged and MP-treated models, immuno-fluorescently stained, was visualized via confocal laser scanning fluorescence microscopy (LSM).

#### Immunofluorescent Staining

Upon completion of the exposures, the models were washed with PBS, and half of the membrane inserts (the remaining half which was not used for real-time RT-qPCR) were fixed in 4% paraformaldehyde (in PBS, v/v) for 15 min, washed three-times with PBS and stored at 4°C until the staining procedure. Then, cells were permeabilized with 0.2% Triton X-100 (in PBS, v/v) for 15 min and washed (three-times with 0.1% w/v BSA in PBS). The models were immersed in a mixture of primary antibodies for 2 h: mouse mature macrophage marker monoclonal antibody (clone eBio25F9; eBioscience^TM^; Thermo Fisher Scientific, Germany) at 5 μg/mL and rabbit anti-CD83 antibody (clone EPR22405, Abcam, Switzerland) at 10 μg/mL in 0.1% BSA in PBS. After washing (three-times with 0.1% BSA in PBS), the models were incubated for 2 h in a mixture of secondary antibodies: 20 μg/mL of goat anti-mouse polyclonal secondary antibody, Alexa 647 conjugated (ab150115, Abcam, United Kingdom), 9 μg/mL goat anti-rabbit DY 488 conjugated (AS09 633; Agisera, Sweden) and 0.66 μM rhodamine-phalloidin (Thermo Fisher Scientific, Switzerland) in 0.1% BSA in PBS. After washing (three-times with 0.1% BSA in PBS), the inserts were incubated for an additional 10 min with 1 μg/mL DAPI (Sigma Aldrich, Switzerland) in 0.1% BSA in PBS. All the staining steps were performed in the dark at room temperature. After staining, the cells were washed with PBS, and half of the membranes were cut with a scalpel into two pieces. For optical analysis, both pieces of each sample were mounted in glycergel (DAKO Schweiz AG, Switzerland) to visualize both sides of the model.

#### Confocal Laser Scanning Microscopy

Visualization of the models was conducted with an inverted LSM Zeiss 710 microscope (Carl Zeiss, Switzerland) equipped with a 40x objective lens (EC Plan-Neofluar 40x/1.30 Oil DIC M27). Representative images (z-stacks) of both apical and basal sides were collected and were further processed using the ImageJ-based software Fiji [ImageJ, NIH, US (Schindelin et al., 2012)].

### Barrier Integrity

The barrier integrity of the multicellular models was assessed via a permeability assay using fluorescein isothiocyanate-dextran solution (70 kDa; FITC-dextran). FITC-dextran (stock at 25 mg/mL in water) and EDTA (stock at 0.5 M in water) solutions were prepared in Hank’s Balanced Salt Solution (HBSS) without Mg and Ca salts at 2 mg/mL and 10 mM, respectively. After exposure, the models were washed with HBSS and placed in 6-well plates containing 2 mL of HBSS in the basal compartment. First, 1 mL of HBSS buffer was added to the apical compartment of untreated, LPS-, and MP-exposed models, whereas 1 mL of 10 mM EDTA solution was added for positive control samples for barrier integrity disruption. Then, 1 mL of a 2 mg/mL FITC-dextran solution was added to the apical compartments of all the samples and incubated for 60 min (dark, 37°C). In parallel, a blank insert without cells but with HBSS and FITC-dextran was prepared as described above. After incubation, membrane inserts were immediately removed from the cell-culture plates. The supernatants of HBSS containing FITC-dextran were collected from the basal compartments with precise collected volumes noted for each sample. Samples were kept in the dark until the measurements. Fluorescence intensity was measured in triplicate in black 96-well plates using a microplate reader (Tristar LB 941, Berthold Technologies; using the following filters setup: λex/λem: 485/535 nm). Results were corrected with the supernatant volumes (i.e., fluorescence intensity per mL) and expressed as relative to the average fluorescence measured in blank samples.

### Cell Viability/Membrane Rupture

Lactate dehydrogenase activity was measured in the basal cell culture medium of exposed models and analyzed in triplicate using the LDH cytotoxicity detection kit, as described for the monoculture experiments. Apical application of 0.2 mL Triton-X (0.2% in sterile-filtered ultrapure water) for 24 h served as a positive control. LDH values are presented as fold increase values relative to untreated cells.

### Statistical Analysis

Statistical analysis was performed using GraphPad Prism 8 software (GraphPad Software Inc., San Diego, CA, United States). Parametric one-way analysis of variance (ANOVA; α = 0.05) with *post hoc* Tukey’s multiple comparison test was used to compare values among the different treatments. Statistically significant values among the treatments are shown as: ^∗^*p* ≤ 0.05; ^∗∗^*p* ≤ 0.01; ^∗∗∗^*p* ≤ 0.001, ^****^p ≤ 0.0001.

## Results

The anti-inflammatory effects of MP were first tested on MDM monocultures with respect to the drug’s priming potential to shift macrophage phenotypes towards the anti-inflammatory subset. In the multicellular human alveolar model, LPS-induced inflammation, triggered from apical and basal sides of the model, is demonstrated, followed by four experimental approaches of pro-inflammation induction and its suppression, mimicking various scenarios of lung inflammation and treatment in clinics.

### Macrophage Phenotype Plasticity Upon Pro- and Anti-inflammatory Treatments

Alveolar macrophages play a pivotal role in both pro- and anti-inflammatory reactions in the lung. Therefore, in this study, a known phenomenon of macrophage phenotype plasticity, i.e., the shift from M1 (pro-inflammatory) to M2 (anti-inflammatory) and vice-versa, in response to their microenvironment was initially tested. MDMs were treated with LPS for the first 24 h, and MP was added for an additional 24 h while LPS was left in the system (Table 1). After the exposure, the secretion of pro-inflammatory cytokines was assessed via ELISA and expression of surface markers via flow cytometry. LPS stimulation induced statistically significant secretion of IL-8 and TNF-α (Figure 1A and Table 2), demonstrating that MDMs exhibit responsiveness to LPS stimulation. In contrast, the expression of the M1 marker CD86 remained unchanged (Supplementary Figure S2). LPS exposure resulted in lower CD206 expression than untreated cells, while a combined IL-4 + IL-13 treatment, which is known to induce M2 polarization, induced its significant upregulation (Figure 1B). Next, we tested whether treatment with MP during the latter half of LPS stimulation mitigates manifestation of M1 polarization. Treatment of inflamed MDMs with MP or vehicle alone did not induce secretion of pro-inflammatory cytokines, nor did it cause a phenotypic shift. In contrast, MP treatment of LPS-stimulated MDMs for 24 h resulted in decreased secretion of IL-8 and TNF-α (Figure 1A), while leaving the expression of CD86 and CD206 unchanged (Figure 1B and Supplementary Figure S2). Thus, MP inhibited the secretion of the tested pro-inflammatory markers (IL-8, TNF-α) but did not reprogram the MDM phenotype. None of the treatments used affected the viability of MDMs (Figure 1C and Supplementary Figure S2a). There are four individual repetitions representing the four blood donors. The gating strategy is presented in the Supplementary Material (Supplementary Figure S1).

**TABLE 2:** A summary of the pro-inflammatory reactions in monocultures and multicellular human lung models.

		*MDMs*			*LPS or MP application/in vivo significance*	*Approach 1*			*Approach 2*			*Approach 3*			*Approach 4*		
							
	*Treatment*	*MP10 Ctrl*	*LPS*	*LPS + MP10*	LPS	*Basal/blood-derived inflammation*			*Apical/inflammation from the airways*			*Apical/inflammation from the airways*			*Basal and apical/strong tissue inflammation*		

Surface marker expression	CD86	0	+	0	MP	*Apical/pulmonary drug delivery*			*Basal/systemic drug administration*			*Apical/pulmonary drug delivery*			*Apical/pulmonary drug delivery*		
Pro-inflammatory mediators secretion	**IL-8**	0	+++	++		−	++	+	−	+	+	*NA*	++	+	*NA*	+	−
	**TNF-α**	0	+++	++		−	+	+	−	++	+		+	+		+	+
	**IL-1β**	*NA*	*NA*	*NA*		−	+	+	−	+	+		+	+		+	+
			**Pro-inflammatory**		***CXCL8***	−	++	−	−	++	−		++	−		++	−
			**gene expression**		***TNF***	−	−	++	−	−	+		+	−		++	−
					***IL1B***	−	0	0	−	++	+		++	0		+	−

### LPS-Induced Inflamed Multicellular Human Lung Model

**FIGURE 2:**
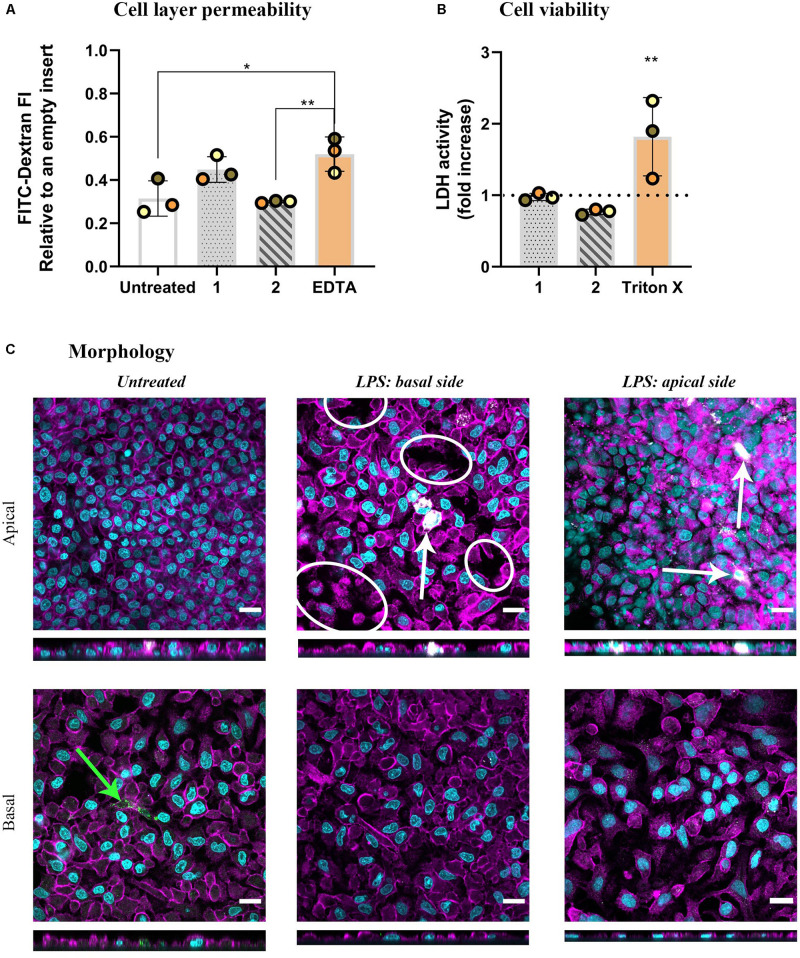
The results of barrier permeability, viability, and morphology assessment of inflamed multicellular models after LPS challenge (1 μg/mL, 48 h) from either basal (approach 1) or apical compartment (approach 2). **(A)** Barrier permeability assessed via FITC-Dextran (70 kDa) permeability assay. The data is presented as fluorescence intensity of FITC-Dextran, measured in the basal compartment, normalized to the values of empty inserts, as the mean of three biological repetitions ± standard deviation. Individual values from biological repetitions are presented as circles, color-coded to represent the data from the same biological repetitions. EDTA (5 mM, 60 min) was used as a positive control for the barrier disruption analysis. **(B)** Cell viability assessed via a membrane rupture assay based on the LDH enzyme released in the cell culture medium of the basal compartment presented as fold increase over untreated cells (the dotted line). Positive control cells were apically exposed to Triton X-100 (0.2%; 24 h). Statistically significant differences among the groups (One-way ANOVA, Tukey’s *post hoc*; α = 0.05) or to untreated cells (LDH assay): *p ≤ 0.05; **p ≤ 0.01. **(C)** Morphology, visualized via confocal laser scanning microscopy of apical and basal sides of the inserts, shown as XY and XZ projections. Immuno-fluorescence labeling: nuclei (cyan), cytoskeleton (magenta), MDMs (mature macrophage marker 25F9; white), MDDCs (CD 83; green). White ellipsoids point out the regions with epithelial barrier disruption. The white arrow denotes MDMs, whereas the green arrow denotes MDDCs. Scale bars are 20 μm.

As the next step, we adapted a more complex lung model to investigate the orchestrated immune cell responses upon exposure to LPS with or without the presence of MP: multicellular models, composed of alveolar epithelial cells (A549) and immune cells (MDDCs and MDMs on basal and apical sides, respectively). The models were challenged with LPS for 48 h from either basal (approach 1) or apical (approach 2) sides. As inflammation in an epithelial tissue often leads to impaired epithelial barrier integrity, the permeability of the model to labeled dextran (70 kDa) was first assessed. LPS challenge from the basal side resulted in increased tissue permeability, demonstrated by the absence of significant differences compared to the positive control (EDTA-treated) models, indicating a disruption of barrier integrity (approach 1). In contrast, apical LPS application (approach 2) did not alter the permeability compared to untreated cells but there was statistical significance observed compared to the positive control models (Figure 2A). Neither of the treatments induced membrane rupture as assessed by LDH release in the basal cRPMI (Figure 2B). The morphology of the epithelial layer on the apical side of the insert was impaired in approach 1 and also slightly after the apical LPS challenge in approach 2. In contrast, the morphology of the basal sides of the inserts, i.e., at the side of MDDCs, was comparable with untreated cells for both approaches (Figure 2C).

**FIGURE 3:**
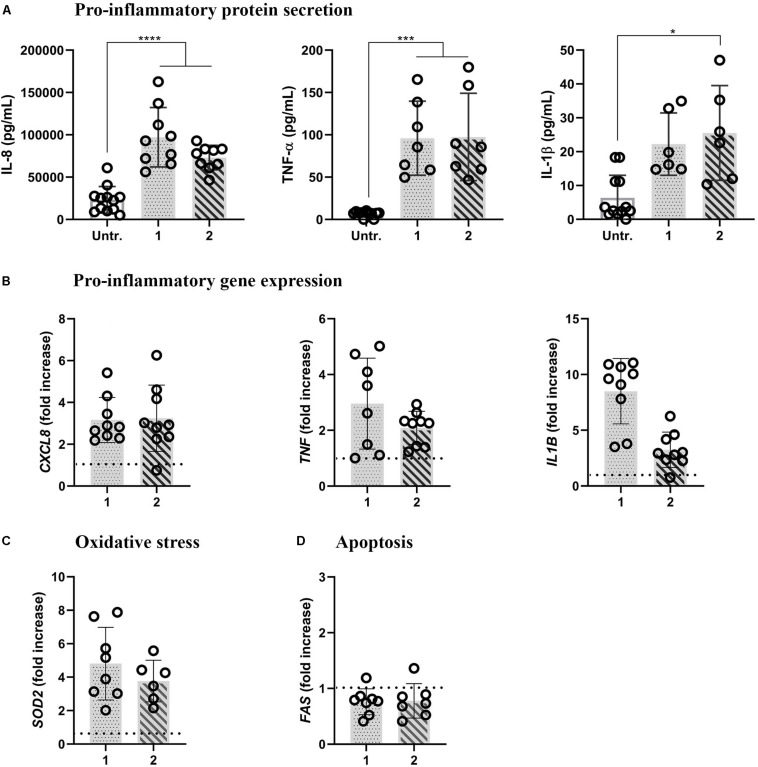
Pro-inflammatory, oxidative stress, and apoptotic reactions in inflamed multicellular models upon LPS challenge (1 μg/mL, 48 h) from either basal (approach 1) or apical compartment (approach 2). **(A)** Secretion of pro-inflammatory mediators IL-8, TNF-α, and IL-1β (upper panel) and **(B)** the respective gene expressions (lower panel). The data for protein secretion are shown in pg/mL, measured in the basal compartment via ELISA. The data on gene expression, assessed via real-time RT-qPCR, is shown as a fold increase of mRNA calculated via the ΔΔCt method, i.e., normalized to the expression of the housekeeping gene GAPDH and the expression of the gene of interest in the untreated samples. The dotted lines denote the mean values of the untreated cells. **(C)** Oxidative stress and **(D)** apoptotic gene expression levels, assessed via real-time RT-qPCR. Statistical analysis for ELISA was performed on pg/mL values using One-way ANOVA (Tukey’s *post hoc*; α = 0.05). For gene expression, statistically significant differences compared to the untreated cells are shown. *p ≤ 0.05; **p ≤ 0.01; ***p ≤ 0.001, ****p ≤ 0.0001. Abbreviations: Untr., untreated models; IL-8, *CXCL8*, interleukin 8; TNF-α, *TNF*, tumor necrosis factor α; IL-1β, *IL1B*, interleukin1β; *SOD2*, superoxide dismutase; *FAS*, Fas cell surface death receptor.

LPS stimulation significantly increased the expression of pro-inflammatory mediators at protein and gene expression levels, irrespective of the approach used. In both approaches, LPS induced the secretion of IL-8, TNF-α, and IL-1β (the IL-1β secretion was statistically significant only in approach 2; Figure 3A). Accordingly, in both approaches, increased mRNA levels were observed compared to untreated cells, with the lowest difference observed for *IL1B* in approach 2 (Figure 3B). LPS-induced oxidative stress was evidenced with the onset of mitochondrial antioxidant superoxide dismutase-2 (*SOD2*) mRNA levels in both approaches (Figure 3C). The absence of pro-apoptotic reactions was evidenced by unaltered *FAS* mRNA expression levels in both the approaches after 48-h LPS treatments (Figure 3D).

### Corticosteroids Suppress Pro-inflammatory Reactions in the Inflamed Model

**FIGURE 4:**
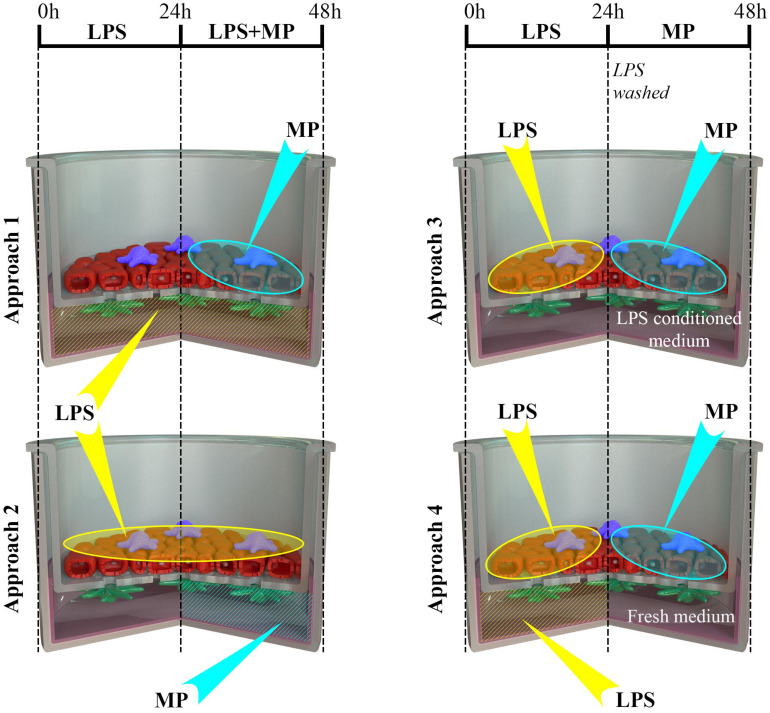
Schematic representation of the four scenarios (approaches 1 to 4) of LPS and MP applications to the multicellular models. Multicellular human lung models, composed of alveolar epithelial cells (A549; red) and human monocyte-derived macrophages (MDMs; blue) and dendritic cells (MDDCs; green), were cultivated at the air-liquid interface conditions (ALI) for 24 h. Subsequently, the models were challenged with LPS (at time 0 h), followed by treatment with MP following four distinct approaches. **Approach 1**: LPS was applied in the basal compartment (1 μg/mL in 3 ml cRPMI), and after the first 24 h, MP was added to the apical side as a thin layer of liquid (i.e., at pseudo-ALI) at 10 or 100 μM, for an additional 24 h. **Approach 2**: LPS was added to the apical compartment at the pseudo-ALI for the first 24 h, and then MP was added in co-exposure from the basal compartment for the next 24 h. **Approach 3**: LPS, applied apically at the pseudo-ALI, was removed after 24 h, and the models were then exposed to the pre-conditioned medium during the MP treatment for an additional 24 h. **Approach 4**: Multicellular models were challenged with LPS both from the apical and basal compartment for 24 h. Then, LPS was removed from both the compartments, the model was washed, and a fresh cell-culture medium was applied during the MP treatment. Abbreviations: LPS, lipopolysaccharide; MP, methylprednisolone.

**FIGURE 5:**
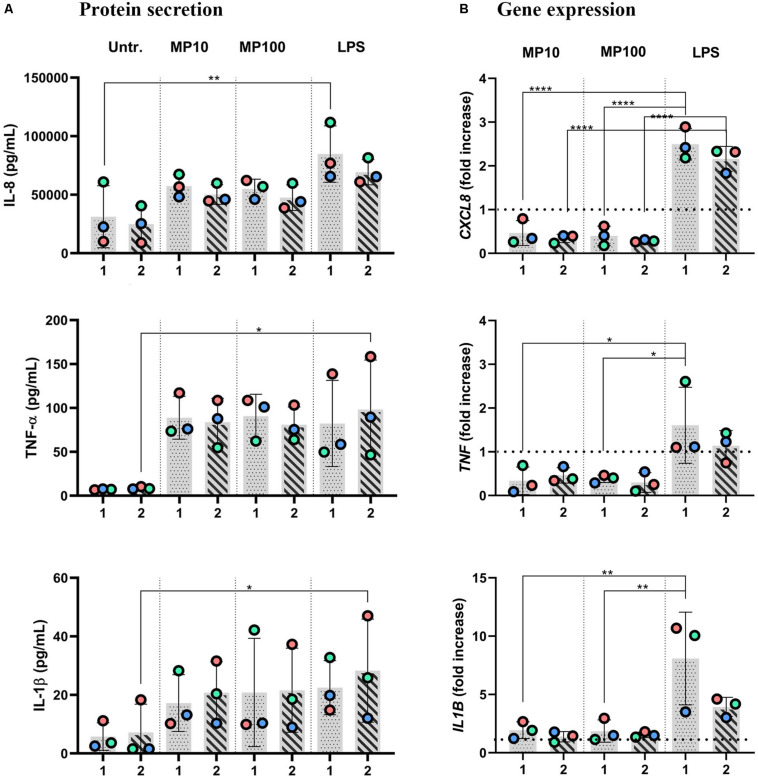
Anti-inflammatory reactions after the addition of MP to inflamed multicellular models in the co-presence of LPS. The *x*-axis represents the number of the approach applied: approaches 1 and 2. Inflamed models were triggered with LPS from either basal (approach 1) or apical (approach 2) compartments for 24 h; then, MP was applied to the opposite compartment for an additional 24 h at 10 or 100 μM. **(A)** Secretion of pro-inflammatory mediators IL-8, TNF-α, and IL-1β and **(B)** the respective gene expressions. The data of protein secretion is shown in pg/mL, measured in the basal compartment via ELISA. The data on gene expression, assessed via real-time RT-qPCR, is shown as fold increase of mRNA calculated via the ΔΔCt method, i.e., normalized to the expression of the housekeeping gene *GAPDH* and the expression of the gene of interest in the untreated samples. Dotted lines denote the mean value of untreated cells. Statistical analysis for ELISA was performed on pg/mL values using One-way ANOVA (Tukey’s *post hoc*; α = 0.05). For gene expression, statistically significant differences to untreated cells are shown. *p ≤ 0.05; **p ≤ 0.01; ***p ≤ 0.001, ****p ≤ 0.0001. Abbreviations: Untr.: untreated models; MP10 and MP100: models treated with LPS (24 h) followed by methylprednisolone at 10 or 100 μM for an additional 24 h; LPS: positive control models, i.e., treated with LPS for 48 h.

**FIGURE 6:**
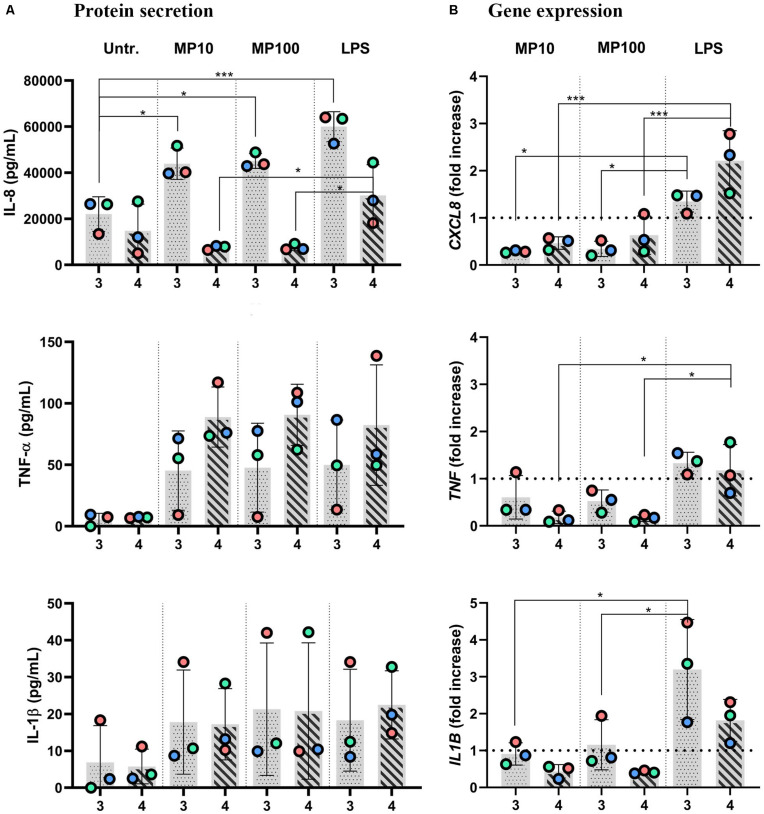
Anti-inflammatory reactions after addition of MP to inflamed multicellular models upon removal of LPS. The *x*-axis represents the number of the approach applied: approaches 3 and 4. Inflamed models were triggered with LPS from the basal compartment (approach 3) or both sides (approach 4) for 24 h. Then, either LPS was removed from the apical side with the cRPMI left in during the apical MP treatment (approach 3) or cRPMI was removed, and inserts were thoroughly washed before MP application (approach 4) for an additional 24 h, at 10 or 100 μM MP. **(A)** Secretion of pro-inflammatory mediators IL-8, TNF-α, and IL-1β and **(B)** the respective gene expressions. The data of protein secretion is shown in pg/mL, measured in the basal compartment via ELISA. The data on gene expression, assessed via real-time RT-qPCR, is shown as fold increase of mRNA calculated via the ΔΔCt method, i.e., normalized to the expression of the housekeeping gene *GAPDH* and the expression of the gene of interest in the untreated samples. The dotted lines denote the mean value of untreated cells. Statistical analysis for ELISA was performed on pg/mL values using One-way ANOVA (Tukey’s *post hoc*; α = 0.05). For gene expression, statistically significant differences to untreated cells are shown. *p ≤ 0.05; **p ≤ 0.01; ***p ≤ 0.001. Abbreviations: Untr., untreated models; MP10 and MP100, models treated with LPS (24 h) followed by methylprednisolone at 10 or 100 μM for additional 24 h; LPS, positive control models, i.e., treated with LPS for 24 h, then washed accordingly and treated with the vehicle instead of MP.

Next, we examined whether the MP treatment of LPS-stimulated multicellular model leads to decreased production of pro-inflammatory factors IL-1 β, IL-8, and TNF-α, following four approaches of LPS and MP application. In approaches 1 and 2, MP treatment of inflamed models was performed in the presence of LPS. LPS and MP were applied either in the basal or the apical compartments (approach 1) or vice-versa (approach 2), respectively (Figure 4; left panel). Alternatively, in approaches 3 and 4, LPS was removed from the system before the application of MP. In approach 3, inflamed models were treated with MP while being in contact with LPS-conditioned medium, whereas the basal compartment was refilled with fresh medium in approach 4 (Figure 4; right panel). At the protein level, there was an overall trend of slightly reduced secretion of pro-inflammatory mediators (Figure 5A). Interestingly, however, the mRNA levels of all three factors were found to have dropped significantly 24 h after the addition of MP in approach 1, which generally induced higher expression of mRNA of the pro-inflammatory factors. A similar trend was observed when we treated the multicellular model using approach 2 (Figure 5B). The trends in pro-inflammatory mediator secretion were also observed in approaches 3 and 4 (Figure 6A), where LPS was removed before the apical application of MP (Figure 4; right panel). Reduced mRNA levels of pro-inflammatory mediators were observed upon exposure to MP for 24 h compared to positive controls (“LPS”; Figure 4), yet this was statistically significant for *CXCL8* in both approaches 3 and 4, *TNF* in approach 4 and *IL1B* in approach 3 (Figure 6B). Of the three mediators, MP treatment decreased the IL-8 level the most. A summary of results regarding pro-inflammatory mediator secretion and mRNA levels, along with the monoculture phenotype-associated endpoints, are presented in Table 2.

**FIGURE 7:**
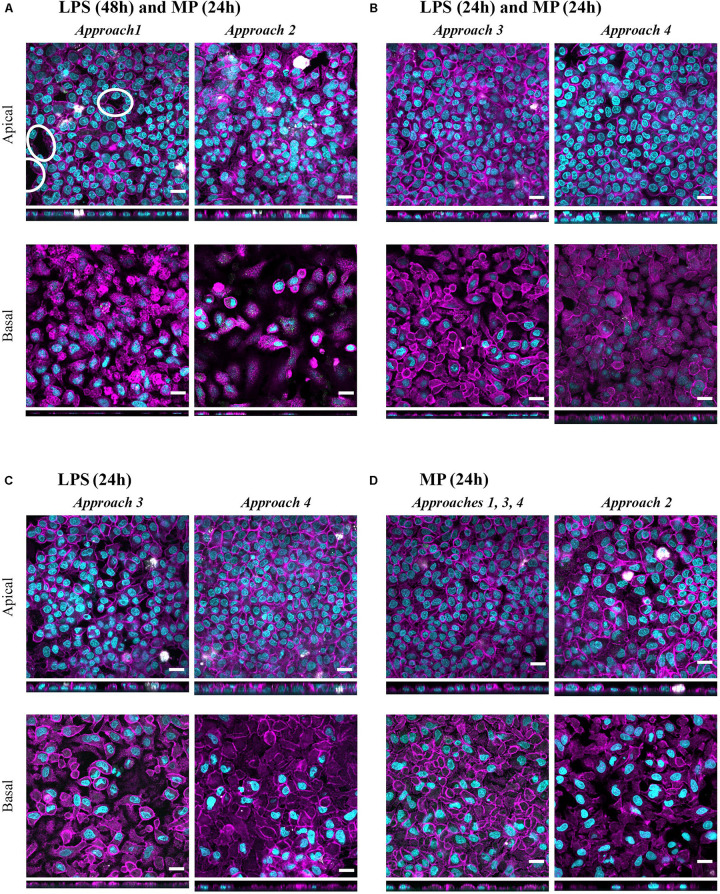
Confocal microscopy images representing the tissue morphology upon exposures to LPS+MP, and LPS or MP alone for 24 h. Multicellular models were treated with **(A)** LPS (24 h + 24 h) and MP at 100 μM from either apical (approach 1) or basal (approach 2) compartments, with **(B)** LPS (24 h) and MP at 100 μM (24 h) corresponding to approaches 3 and 4, respectively **(C)** LPS (24 h) corresponds to the positive controls in approaches 3 and 4. **(D)** MP control tissues, treated only with MP at 100 μM (24 h) from either apical (approaches 1, 3, 4) or basal (approach 2) compartments. Confocal laser scanning microscopy of apical and basal sides of the inserts, shown as XY and XZ projections. Immuno-fluorescence labeling: nuclei (cyan), cytoskeleton (magenta), MDMs (mature macrophage marker 25F9; white), MDDCs (CD 83; green). White ellipsoids point out the regions with epithelial barrier disruption. Scale bars are 20 μm.

In all four approaches, the resolution of LPS-induced oxidative stress that had occurred in inflamed models (Supplementary Figure S3) was observed, as evidenced by increased levels of the mitochondrial antioxidant superoxide dismutase-2 (*SOD2*) compared to the positive control models (“LPS”; Figure 4). These trends were more pronounced in multicellular models treated through approaches 1 and 2, where LPS was applied for 48 h (Supplementary Figure S3). None of the treatments exerted notable effects on apoptotic gene expression levels (*FAS* mRNA levels; Supplementary Figure S3b). Morphology of the apical epithelial layer remained mostly unaffected after all of the treatments (Figure 7), similar to what was observed for untreated cells (Figure 2C); only in approaches 1 and 2 did the cell layer integrities appear to be slightly disrupted (Figure 7A). The morphological appearance remained unaltered after the apical or basal application of MP alone (Figure 7D) relative to the untreated counterparts (Figure 2C). These healthy models (in the absence of LPS) treated with MP alone served as a control to rule out the potential effects of MP alone. Exposure to MP at 100 μM both apically and basally did not induce changes in barrier integrity and cell viability (Supplementary Figures S4a,b). Levels of secreted IL-8, TNF-α, and IL-1β were lower than in the untreated cells, although the differences were not statistically significant. Similarly, the mRNA content was slightly reduced in all MP-treated samples, with the most pronounced effect observed for *CXCL8* (Supplementary Figure S4c). MP treatment resulted in a slight reduction in *SOD2* mRNA levels in approaches 1 and 2 (Supplementary Figure S4d) while leading to a modest increase in the mRNA level of the apoptotic gene *FAS* (Supplementary Figure S4e). Given the small degree of differences, however, we considered these effects as biologically insignificant.

## Discussion

In this study, we present a multicellular human lung model and the resolution of LPS-induced inflammation *in vitro* using a corticosteroid drug, MP. Four experimental approaches to pro-inflammation induction and its suppression are presented, mimicking various scenarios of lung inflammation and treatment in clinics. As alveolar macrophages are the main actors in the induction and resolution of pro-inflammatory reactions in the tissue, we discuss the anti-inflammatory effects of MP considering its priming potential to shift macrophage phenotypes towards the anti-inflammatory subset and with the potential to reduce pro-inflammatory reactions *in vitro* (Table 2).

### Pro-inflammatory Reactions and Impaired Alveolar Barrier Integrity in Inflamed Models

Initially, we used a previously established multicellular model of the human alveolar epithelium (Bisig et al., 2019; Barosova et al., 2020) to induce an inflammatory model via a 48-h challenge with LPS. The LPS was applied either from the basal side of the models simulating a systemically derived alveolar inflammation or from the apical side mimicking the source of the tissue inflammation derived from inhaled stimulants. Microbial products such as LPS are a widely used pro-inflammatory stimulus in *in vitro* assays because of their prominent activity to stimulate innate immune responses (Rosadini and Kagan, 2017). The primary mechanism of LPS-induced response begins through its action towards the toll-like receptor 4 (TLR-4), which is present on both of the immune cell types within the model, i.e., macrophages and dendritic cells (Visintin et al., 2001; Fekete et al., 2012). Upon activation of TLR-4, the onset of the nuclear factor kappa B (NF-kB) signaling pathway results in an increase in the secretion of pro-inflammatory cytokines and chemokines, such as the main actor of central inflammation, TNF-α, and interleukins, e.g., IL-1 (Beutler, 2000; Nova et al., 2019). As LPS-induced macrophage phenotype shifts towards M1 have been observed (Wang et al., 2014; Lu et al., 2018; Orecchioni et al., 2019), we employed LPS as the pro-inflammatory stimulus in both the macrophage monocultures and multicellular experiments.

The onset of pro-inflammatory reactions of both the mono- and multicellular models upon LPS challenge can be explained by the high expression of TLR-4 in mononuclear cells (Beutler and Rietschel, 2003; Lawrence and Natoli, 2011), i.e., MDMs and MDDCs. Additionally, the CD14 receptor present in these cells is also involved in the extracellular binding of LPS, which contributes to the initiation of the NF-kB signaling cascade (Beutler, 2000; Lu et al., 2008). Stimulation of MDM monocultures with LPS induced secretion of pro-inflammatory mediators (IL-8, TNF-α) depleted the mannose receptor (CD206) expression (Figure 1) and slightly increased CD86 expression, which are established markers of M2 and M1 activation, respectively (Lawrence and Natoli, 2011; Smith et al., 2016). It is important to note that the classical division into distinctive macrophage phenotypes has been questioned in multiple studies, which have proposed that cells adopt a mixed phenotype depending on the stimuli present in their microenvironment, rather than polarization into two distinctive phenotypes (Smith et al., 2016; Atri et al., 2018; Orecchioni et al., 2019). M-CSF, used for monocytes differentiation into macrophages, is also very effective in polarizing macrophages towards the M2 phenotype (Fleetwood et al., 2007; Orecchioni et al., 2019). In fact, by the time we removed the M-CSF from the MDM differentiation culture, the cells were already skewed towards the M2 phenotype, and the addition of LPS might not have been a strong enough stimulus to trigger surface marker expression alterations. The addition of interferon-γ to LPS likely has a synergistic effect of facilitating skewing towards the M1 phenotype along with LPS, as was observed in parallel experiments (data not shown) and in previous reports (Müller et al., 2017; Orecchioni et al., 2019). In this study, however, we focused on LPS-challenged MDMs to compare the onset and suppression of pro-inflammatory reactions between a 2D monoculture setting and more realistic exposures in 3D models.

It should be noted that in *in vivo* settings, macrophages and dendritic cells both respond to microbial danger signals by secretion of inflammatory cytokines and interact with the alveolar epithelial cells as well as one another (Banchereau and Steinman, 1998; Beutler and Rietschel, 2003; Gordon, 2003; Fekete et al., 2012; Cook and MacDonald, 2016). Therefore, *in vitro* multicellular models simulate *in vivo* settings more faithfully than do 2D monocultures (Fitzgerald et al., 2015), as they enable an interplay between the three relevant cell types of alveolar epithelial tissue to take place *in vitro* (Rothen-Rutishauser et al., 2005; Blank et al., 2007). In addition to the immune cells in the model, epithelial cells themselves are also responsive to a pro-inflammatory stimulus, such as LPS (Guillot et al., 2004). LPS stimulation of monocultures of A549 cells results in increased pro-inflammatory marker secretion (IL-8, TNF-α) and the respective gene expression (CXCL8 and TNFA). However, the pro-inflammatory response is significantly lower as compared to that in the co-culture model including immune cells (Bisig et al., 2019; Barosova et al., 2020). Indeed, we observed more pronounced pro-inflammatory reactions in the inflamed multicellular alveolar models, regardless of the side of LPS application, relative to their untreated counterparts (Figure 3). However, it is important to note that stressor-induced inflammation in a tissue is a highly complex process involving a combination of events. According to the recently proposed adverse outcome pathway (AOP) framework (Villeneuve et al., 2018), the pro-inflammatory reactions observed in our experiments can be classified as part of the second general key event (KE2): increased pro-inflammatory mediators and a loss of the alveolar barrier integrity (Villeneuve et al., 2018) due to tight junction disruption (Chignard and Balloy, 2000; Eutamene et al., 2005). The latter was observed in the multicellular model upon 48 h of basal LPS exposure (approach 1; Figure 2A). Reduced barrier integrity upon basal LPS challenge (approach 1) was also confirmed by the change in the morphological appearance of the multicellular models. In essence, distinctive patches of ruptured tissue were observed on the apical side of the inserts, whereas the confluency of the apical cell layer in approach 2 was comparable to untreated cells (Figure 2C). As we have shown earlier that tight junctions in A549 appear irregularly (Hilton et al., 2019) we did not perform a tight junction staining but assessed the barrier integrity by a permeability assay using FITC-dextran. This suggests that the cytokines released by the MDDCs, present on the basal side of the multicellular model, may affect tight junctions in the epithelial barrier via the release of pro-inflammatory cytokines (Prakash et al., 2014; Agrawal, 2017) and it seems that MDMs are more effective at resolving the inflammation. Consequently, the epithelial permeability is enhanced, which, in *in vivo* settings, allows for infiltration of immune cells at the site of infection/inflammation (Martin et al., 1997). Our observations thus suggest that our inflamed multicellular model can mimic a combination of inflammation-distinctive features in the human lung tissue.

Oxidative and apoptotic reactions are other hallmarks of LPS-induced tissue inflammation (Mukhopadhyay et al., 2006; Ma et al., 2010; Nova et al., 2019). Expression of an oxidative stress-related gene, a mitochondrial antioxidant, manganese-dependent superoxide dismutase (MnSOD) SOD2 was upregulated in the LPS-challenged multicellular models as compared to untreated cells (Figure 2C). This result is in line with observations of LPS-challenged monocytes, macrophage cell lines, and primary cells *in vitro*, where *SOD2* upregulation is associated with the NF-kB pathway activation (Sharif et al., 2007; Widdrington et al., 2018). Another reason for the increased SOD2 expression can be attributed to MDDCs (Jin et al., 2010). In our untreated multicellular models, MDDCs induced by GM-CSF and IL-4 remain in their immature state, with the ability to take up and process antigen (Fekete et al., 2012). When the models are stimulated with LPS, MDDCs undergo their maturation process, which is accompanied by a 28-fold upregulation of *SOD2* in 24 h (Jin et al., 2010). Activation of the Fas-mediated apoptosis pathway has been reported to play a key role in inflammation-induced damage of the alveolar epithelium (Ma et al., 2010). In our study, we did not observe a statistically significant upregulation of *FAS* mRNA levels, nor was there any increase in cell membrane rupture as a measure of cell viability in either multicellular models or monoculture (Figures 1C, 2B, and Supplementary Figure S2a). This was nevertheless expected, as we had chosen a sub-cytotoxic LPS concentration based on our previous studies (Bisig et al., 2019).

### Anti-inflammatory Reactions Induced With Corticosteroids

In the next step of the study, we treated the inflamed monocultures and multicellular models with a known anti-inflammatory therapy, corticosteroids. Corticosteroids exert anti-inflammatory activity through their binding to corticosteroid hormone receptors, and this process operates via several molecular mechanisms (Barnes, 2011). When corticosteroids bind to glucocorticoid receptors (GR), which are expressed in almost all cell types, the resulting GR-glucocorticoid complex can take either of two paths: (i) the activated GR complex upregulates the expression of anti-inflammatory proteins the nucleus or (ii) the GR complex represses the expression of pro-inflammatory cytokines and chemokines, or their mRNA degradation (Barnes, 2011).

In the monoculture experiments, we investigated the effect of MP on the priming of inflamed MDMs towards the anti-inflammatory M2 phenotype, as has been reported for MP and other corticosteroids in ex vivo and *in vitro* human and *in vivo* animal studies (Frankenberger et al., 2005; Tu et al., 2017; Desgeorges et al., 2019; Xie et al., 2019). We observed decreased IL-8 and TNF-α secretions and CD86 expression, together with increased CD206 expression, relative to that of LPS-treated cells, albeit without statistical significance among the two groups (Figure 1 and Supplementary Figure S2; Table 2). Thus, these findings suggest that MP inhibits the secretion of pro-inflammatory mediators but does not appear to reprogram the MDM phenotype. A possible reason for the absence of more pronounced differences in cellular phenotypes upon MP treatment in our study is that LPS was not removed from the medium before the MP treatment in order to make the environment comparable with approaches 1 and 2 of the multicellular system. However, as discussed above, the M1/M2 paradigm should not be perceived as two distinctive sub-populations, but rather as a continuum of macrophage polarization states (Edholm et al., 2017; Tu et al., 2017). Therefore, our findings on the increased percentage of M2-associated surface markers accompanied by decreased M1 markers present a valid observation, and suggest that MP helped to balance the macrophage population by shifting them towards the anti-inflammatory phenotype.

Furthermore, in multicellular models, the efficiency of MP in dampening pro-inflammatory reactions was confirmed in models in all of the four different experimental settings applied herein. Overall, the reduction of inflammation compared to the positive controls (“LPS”) was comparable among the four approaches (summarized in Table 2). Consistent trends were observed in the resolution of LPS-induced inflammation by MP (Figure 5). Analogous to the monoculture data (Figures 1A,B), the absence of a potent anti-inflammatory effect can be attributed to the simultaneous presence of LPS during the anti-inflammatory treatment. Our findings suggest that the multicellular model recapitulated the effect of MP treatment observed in the monocellular culture of MDMs (summarized in Table 2), but potentially with delayed kinetics. In fact, the *in vivo* immunosuppressive properties of corticosteroids, including MP, are primarily associated with their influence on macrophages and T lymphocytes (Coutinho and Chapman, 2011), yet their immunosuppressive effect on dendritic cells and alveolar epithelial cells should not be neglected (Zach et al., 1993; Piemonti et al., 1999; Rozkova et al., 2006; Klaßen et al., 2017). Therefore, it is vital to use an immunocompetent multicellular lung model, i.e., one including immune cells along with epithelial cells, to achieve a realistic assessment of the anti-inflammatory and immunosuppressive actions of corticosteroids. Namely, by applying LPS in the basal compartment and the drug at the apical side (approach 1), we mimicked a systemic inflammation caused by blood-derived danger signals and an anti-inflammatory treatment via inhalation. Vice versa, in approach 2, alveolar inflammation was mimicked by applying LPS apically and MP in the basal compartment, simulating an intravenous steroid therapy (Figure 4). Our findings of reduced pro-inflammation in approaches 1 and 2 suggest that MP can be used as an anti-inflammatory drug to model corticosteroid therapy of severe alveolar inflammation in the absence of other treatments that facilitate the resolution of inflammation (such as an antibiotic-based bacteria elimination).

Having demonstrated that a reduction of pro-inflammatory reactions can be assessed *in vitro* in the presence of the stimulus, we developed this test system further by reducing the inflammation. With these *in vitro* conditions, we simulated *in vivo* settings involving pre- or concurrent therapy with agents other than corticosteroids (approaches 3 and 4; Figure 4). Similar trends in the reduction of secretion of the pro-inflammatory mediators and their respective mRNA levels (Figure 6) were observed as in the MP treatments following the previous two approaches (Figure 5 and Table 2). The mildly disrupted cell layer integrities in approaches 1 and 2 (Figure 7A) can be attributed to the extended LPS challenge (48 vs. 24 h), as similar disintegration of the apical cell layer was observed for the positive control models, i.e., those treated with LPS for 48 h (Figure 2C), and less disintegration was observed after 24 h (Figure 7B). Overall, inflamed models treated with MP presented higher cell-layer integrity than positive control samples (LPS), especially those following approach 3 (Figures 2C, 7). This was expected, however, as MP has been demonstrated to lead to complete resolution of *in vivo* and *in vitro* mechanical and histological lung alterations in mice (Silva et al., 2009). Furthermore, a minimal reduction of pro-inflammatory reactions in healthy tissues was observed upon treatment with MP for all the pro-inflammatory mediators (Supplementary Figure S4c). A possible explanation for this is that the assembly of the immunocompetent multicellular human lung model itself triggers a certain amount of pro-inflammatory response, which can then be further reduced with an anti-inflammatory drug. This is indeed consistent with the common side effects of steroid drugs, which can also affect untargeted, healthy cells and tissues (Waljee et al., 2017).

We observed high variations, especially in the cytokine secretion data upon treatment with MP (Figures 5A, 6A, Supplementary Figure S4c). This can be explained by biological variations of the immune cell source. First, for each experiment, i.e., for each biological replicate, human blood-derived monocytes were isolated from a different blood donor. Second, the medical history of blood donors cannot be revealed due to ethical reasons; thus, it was unknown whether the patient had an ongoing inflammation and/or treatment at the time of blood sampling. Therefore, we consider that the high variations in our results reflect the variability in donors and not the poor robustness of our model.

The anti-inflammatory activity of MP appeared to be independent of the doses tested. In approaches 1, 3, and 4, MP was applied at the pseudo-ALI in concentrations of 10 and 100 μM, whereas in approach 2, MP was diluted with 1.2 mL of cRPMI, with the same final concentrations of 10 and 100 μM. We set two main criteria for the development of our *in vitro* system: (i) the pro-inflammatory reactions fall within a comparable range irrespective of the side of LPS application (as explained under inflamed model) (Bisig et al., 2019; Barosova et al., 2020), and (ii) MP is applied at a clinically relevant dose (Vichyanond et al., 1989) which is high enough to promote anti-inflammatory reactions in an *in vitro* system without causing adverse effects on the tissues. Overall, the concentrations tested in the study (10 or 100 μM) are in the range of MP concentrations evaluated in human bronchioalveolar fluid upon intravenous drug administration, i.e., 2000 ng/mL [corresponding to 5.2 μM (Vichyanond et al., 1989)] and are in line with the doses tested in the *in vivo* and *in vitro* study of the effects of MP on mice macrophage polarization (Tu et al., 2017).

### Clinical Significance

In a clinical setting, MP is predominantly administered intravenously or orally (Kinoshita et al., 2017; Meduri et al., 2018), while other corticosteroid drugs, such as budesonide and formoterol, have proven clinical benefits when inhaled (Barnes, 2011). However, the aim of this study was not to evaluate the action of MP by itself but rather to show the responsiveness of the model to corticosteroid treatment *in vitro*. The purpose of this study was to develop a versatile *in vitro* system to test newly developed drugs, including nanoparticle-based candidates, for the treatment of lung inflammation.

In all of the tested approaches to LPS and MP applications, an efficient reduction of pro-inflammatory reactions was observed at both the protein secretion and gene expression levels (Table 2). The authors recommend assessment of the suppression of pro-inflammatory reactions at the gene expression level for inclusion in the endpoint assessment because the decrease in these reactions was more evident than the changes in the secretion of pro-inflammatory factors. In particular, IL-8 (*CXCL8*) yielded the most significant differences among the tested markers (Table 2). Our model provides a flexible choice of the scenarios to test novel drug candidates, depending on the research question. For example, if testing the efficiency of a systemically administered drug is the aim, approach 2 can be employed (Figure 4). This approach also showed the most pronounced reduction of pro-inflammatory reactions at both protein secretion and gene expression levels (Table 2). In contrast, to test candidates for pulmonary drug delivery, approaches 1, 3, and 4 can be followed. Furthermore, a decrease in barrier integrity was observed for the basal application of LPS for 48 h (approach 1). Therefore, where the AOP framework for inflammation and fibrosis (Villeneuve et al., 2018; Halappanavar et al., 2020) is applied, the use of approach 1 is recommended. When a newly developed therapy is envisaged in a combined treatment with, for example, antibiotics, approach 3 or 4 can be employed to test the anti-inflammatory activities of the candidate when the source of inflammation (bacteria) is removed from the microenvironment. Another advantage of the multiple approaches adopted in our study is that the action of the tested drug in respiratory and systemic delivery models can be compared using approaches 1 and 2, respectively. The advantage of creating such a model is that the interplay between the representative cell types is now in an *in vitro* human lung setting, which successfully mimics several key aspects of an *in vivo* setting.

Depending on the stimulus of interest and the research question, additional cell types can also be added to the model, or part of the model elements can be replaced with a relevant type. For the purpose of this study to showcase the responsiveness of the model, A549 have been selected as the epithelial cell type as they are the most widely used cell lines in human respiratory research. An important feature of A549-based model is that surfactant is secreted at the apical surface when cultivated at the air-liquid interface, a key feature to recapitulate *in vivo* conditions in the model. The A549 cell model also has been used to assess the biokinetic distribution of apically administered liposomal Ciclosporin A, and the data was in agreement with the clinically observed pharmacokinetics profile (Schmid et al., 2017). Thus, this model offers a reliable and relevant *in vitro* platform to study the effects of aerosolized substances. Furthermore, depending on the research question and availability of cells the epithelial cells can be replaced for instance with bronchial cells such as the bronchial cell line 16HBE14o-, or with primary alveolar type I-like cells (Lehmann et al., 2011). Importantly, the choice of immune cells is also versatile. Epithelial cells can be co-cultured with other immune cells, such as natural killer cells (Roth et al., 2017). Immune cells such as macrophages can be replaced with differentiated macrophage-like cells (THP-1), and mast cells and/or endothelial cells can be included in similar models (Klein et al., 2013). Additionally, inclusion of polymorphonuclear cells, such as neutrophils, is foreseen, which would, for instance, enable exploration of neutrophil transepithelial migration. However, the importance of other barriers and immune cells needs to be explored in future studies.

An interesting future application of the presented model could be to compare the responsiveness of the model triggered with LPS with the reactions upon stimulation with other inflammatory agents derived from organic dust, such as mold spores (mold antigens). The latter model resembles, for example, a specific respiratory syndrome such as hypersensitivity pneumonitis or extrinsic allergic alveolitis (Riario Sforza and Marinou, 2017) where MP has been used as one of the main treatment drugs (Buchvald et al., 2011).

In conclusion, we have established an *in vitro* human 3D multicellular alveolar epithelial model that is responsive to both pro-inflammatory stimulation and anti-inflammatory treatment. Responses of the model correlated well with phenotypic shifts of macrophages, highlighting their importance in the faithful reproduction of *in vivo* settings. Supported by the use of permeable membranes, our new model also enables the assessment of the permeability of agents through the barrier. Further development is aimed at coupling our multicellular human lung models with an aerosolization system to assess the efficiency of nebulized compounds. Given that a large number of researchers are currently exploring novel anti-inflammatory formulations, and in particular nanoparticle-based candidates intended for administration via inhalation, we anticipate that our “inflammable” human lung model will serve as a versatile and realistic toolkit for efficient evaluation of the efficiency and safety of various drug candidates.

## Data Availability Statement

The datasets presented in this study can be found in online repositories. The names of the repository/repositories and accession number(s) can be found below: Zenodo [https://zenodo.org] under the doi: “10.5281/zenodo.3776486” and the flow cytometry raw data in the Flow repository under the ID: “FR-FCM-Z2KE” [https://flowrepository.org].

## Ethics Statement

The work involving primary monocytes isolated from human blood was approved by the committee of the Federal Office for Public Health Switzerland (reference number: 611-1, Meldung A110635/2) for the Adolphe Merkle Institute. Written informed consent for participation was not required for this study in accordance with the national legislation and the institutional requirements.

## Author Contributions

BD designed and planned the study, carried out majority of the experiments, analyzed and interpreted the data, and finally drafted the manuscript. BK and ET supported the planning and execution of the tissue culture experiments, and BK also supported the writing process of the main text. HB assisted with monocyte isolation and multicellular model experiments. JA provided expert knowledge on flow cytometry. MS provided lab support with the real-time RT-PCR experiments. AP-F and BR-R were involved in the planning and technical advisory of the study and critically revised the manuscript draft for important intellectual content and approved publication of the content. All authors contributed to the article and approved the submitted version.

## Conflict of Interest

The authors declare that the research was conducted in the absence of any commercial or financial relationships that could be construed as a potential conflict of interest.
