# Supplementary material for: An Inflamed Human Alveolar Model for Testing the Efficiency of Anti-inflammatory Drugs in vitro
Source: Front Bioeng Biotechnol. 2020 Aug 21;8:987. doi: 10.3389/fbioe.2020.00987 (PMC7471931; doi:10.3389/fbioe.2020.00987)
Supplement: Supplementary file 1 [file Data_Sheet_1.pdf]

SUPPLEMENTARY MATERIAL

1 Materials and methods

1.1 Flow cytometry: gating strategy

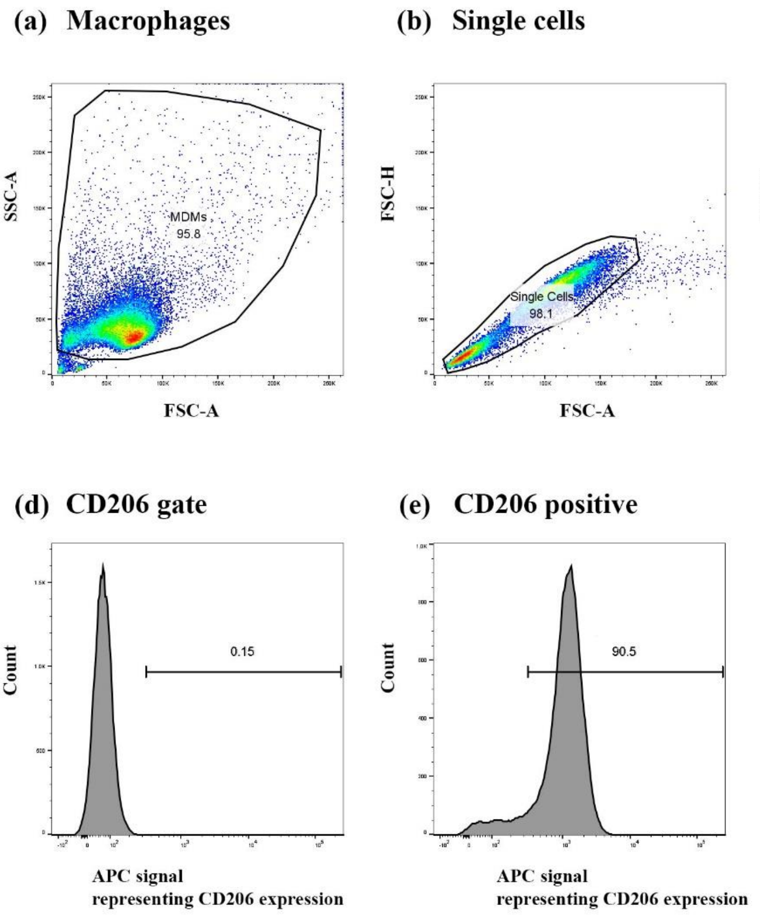

Figure S1: Gating strategy used to determine the median fluorescence intensity of CD86 and CD206 surface markers in human monocyte-derived macrophages (MDMs).

First, the gating was set considering the specific characteristics of the forward- and side-scatters (FSC and SSC), i.e., representing the cell size and granularity, respectively, to distinguish (a) viable macrophages from cell debris and (b) FCS-A/FCS-H to exclude events that could represent more than one cell. DAPI was used as the live/dead discriminator to the gate (c) DAPI negative, which represents the fraction of viable cells within the sample analyzed. Then, the fluorescent signature of fluorescence minus one control (i.e., minus each tested marker; FMO) samples were taken into consideration for each of the tested surface markers (CD206, CD86). (d) An example of FMO-CD206 gating, applied to (e) the stained samples (an example of untreated MDMs is shown). Y-axes in the histograms (c, d, e) represent the count of cells, and X-axes the compensated (c) DAPI and (d and e) APC-CD206 signals. The same gating was applied for the CD86 marker. Results were presented as the median fluorescence intensity (MFI) of the gated live single cell population. All analysis shown was performed using FlowJo (Version 10, TreeStar, USA).

1.2 Real-time RT-qPCR: primer sequences

Table S1: GenBank accession numbers of the analyzed genes and sequences of the primers used for real-time PCR

Gene	GenBank accession	Direction	Primer sequence
GAPDH	NC_000012	Forward	AAC AGC CTC AAG ATC ATC AGC
		Reverse	GGA TGA TGT TCT GGA GAG CC
CXCL8	NM_000584	Forward	CTG GCC GTG GCT CTC TTG
		Reverse	CCT TGG CAA AAC TGC ACC TT
TNF	NM_000594	Forward	CCC AGG GAC CTC TCT CTA ATC A
		Reverse	GCT ACA GGC TTG TCA CTC GG
IL1B	NM_000576.2	Forward	ACG ATC ACT GAA CTG CAC GC
		Reverse	TGT TGC TCC ATA TCC TGT CCC
SOD2	NM_000636.2	Forward	CTG CTG GGG ATT GAT GTG TGG
		Reverse	TGC AAG CCA TGT ATC TTT CAG T
FAS	NM_000043	Forward	AGC TTG GTC TAG AGT GAA AA
		Reverse	GAG GCA GAA TCA TGA GAT AT

2 Results

2.1 Supporting data for monoculture experiments: viability, CD86 expression, signal intensity histograms

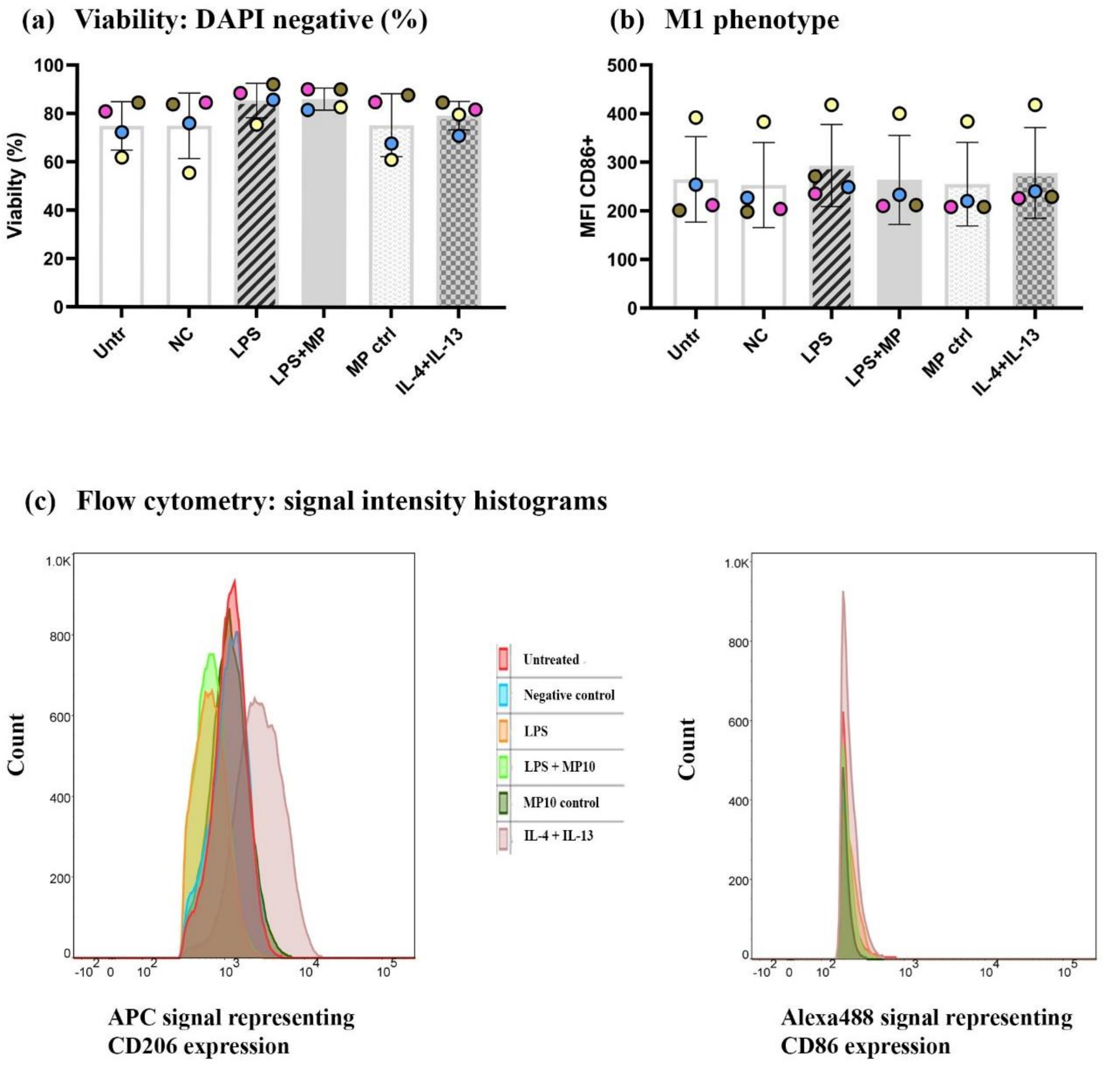

Figure S2: Macrophage monocultures upon exposure to pro- and anti-inflammatory stimuli. (a) Cell viability of MDMs associated with each treatment, assessed via flow cytometry, based on DAPI negative gating, expressed as percentage of all single macrophage cells. (b) Expression of the CD86 surface marker (median fluorescence intensity), denoting M1 phenotype, assessed via flow cytometry analysis. (c) Overlays of CD206 (referring to Figure 1 in the main manuscript) and CD86 intensity histograms, representing the number of counts in each treated group. Representative samples are shown. **Abbreviations:** MFI: median fluorescence intensity; CD86: cluster of differentiation 86; IL-4+IL-13: interleukins 4 and 13, both applied at 20 ng/mL for 48 h.

2.2 Effect of methylprednisolone (MP) on oxidative stress and apoptotic reactions in inflamed models

(a) Oxidative stress

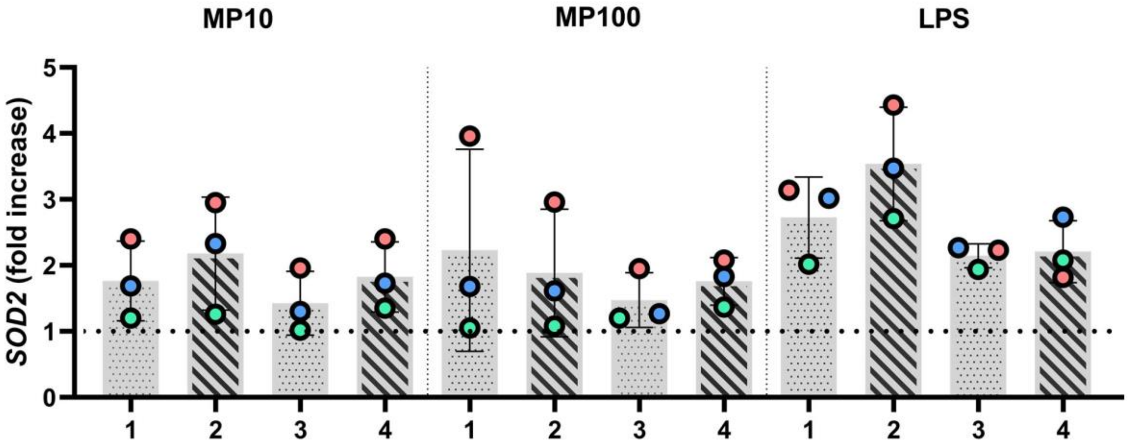

(b) Apoptosis

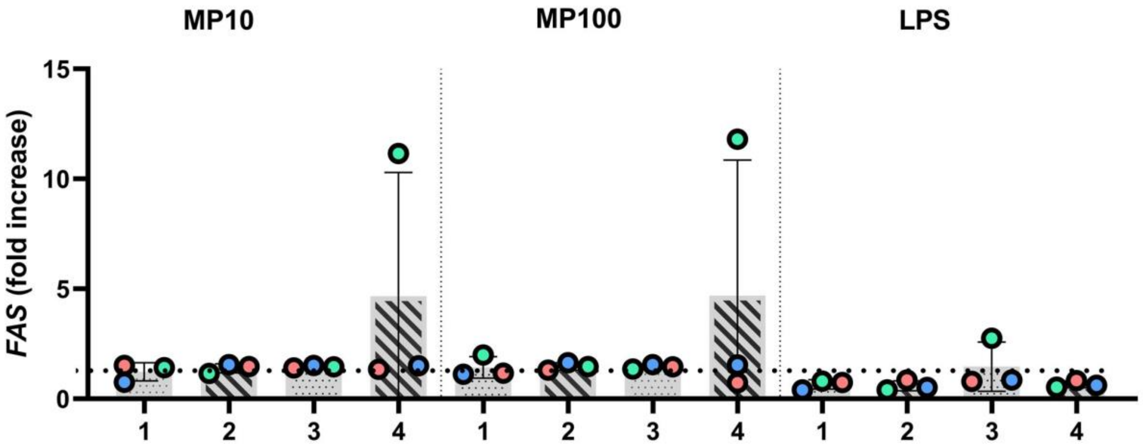

Figure S3: Oxidative stress and apoptotic reactions in inflamed multicellular human lung models treated with MP.

Expression of (a) oxidative stress and (b) apoptotic genes, assessed via real-time RT-qPCR. The data is shown as the fold increase of mRNA calculated via the ΔΔCt method, i.e., normalized to the expression of the housekeeping gene GAPDH and the expression of the gene of interest in the untreated samples. Dotted lines denote the mean value of untreated cells. **Abbreviations:** LPS: lipopolysaccharide; MP: methylprednisolone; 1: approach 1, co-presence of LPS and MP (LPS basal compartment (48 h), MP apical (24 h); 2: approach 2, co-presence of LPS and MP (LPS apical compartment (48 h), MP basal (24 h); 3: approach 3, LPS applied apically for 24 h, then LPS removed, with LPS-conditioned medium remaining in the system during the treatment with MP (24 h); 4: approach 4, inflamed tissues were triggered with LPS from the basal and apical compartment for 24 h, the tissues were washed and fresh medium was applied during the MP treatment (24 h); MP10 and MP100: models treated with LPS (24 h) followed by methylprednisolone at 10 or 100 µM for an additional 24 h; LPS: models exposed to LPS for 48 h from either the basal (approaches 1, 3, 4) or apical compartment (approach 2; 200 µL at pseudo-air-liquid interface). Genes: *CXCL8*: interleukin 8; *TNF*: tumor necrosis factor α; *IL1B*: interleukin1β; *SOD2*: superoxide dismutase; *FAS*: Fas cell surface death receptor.

2.3 Effects of methylprednisolone (MP) on healthy tissues, i.e., in the absence of LPS

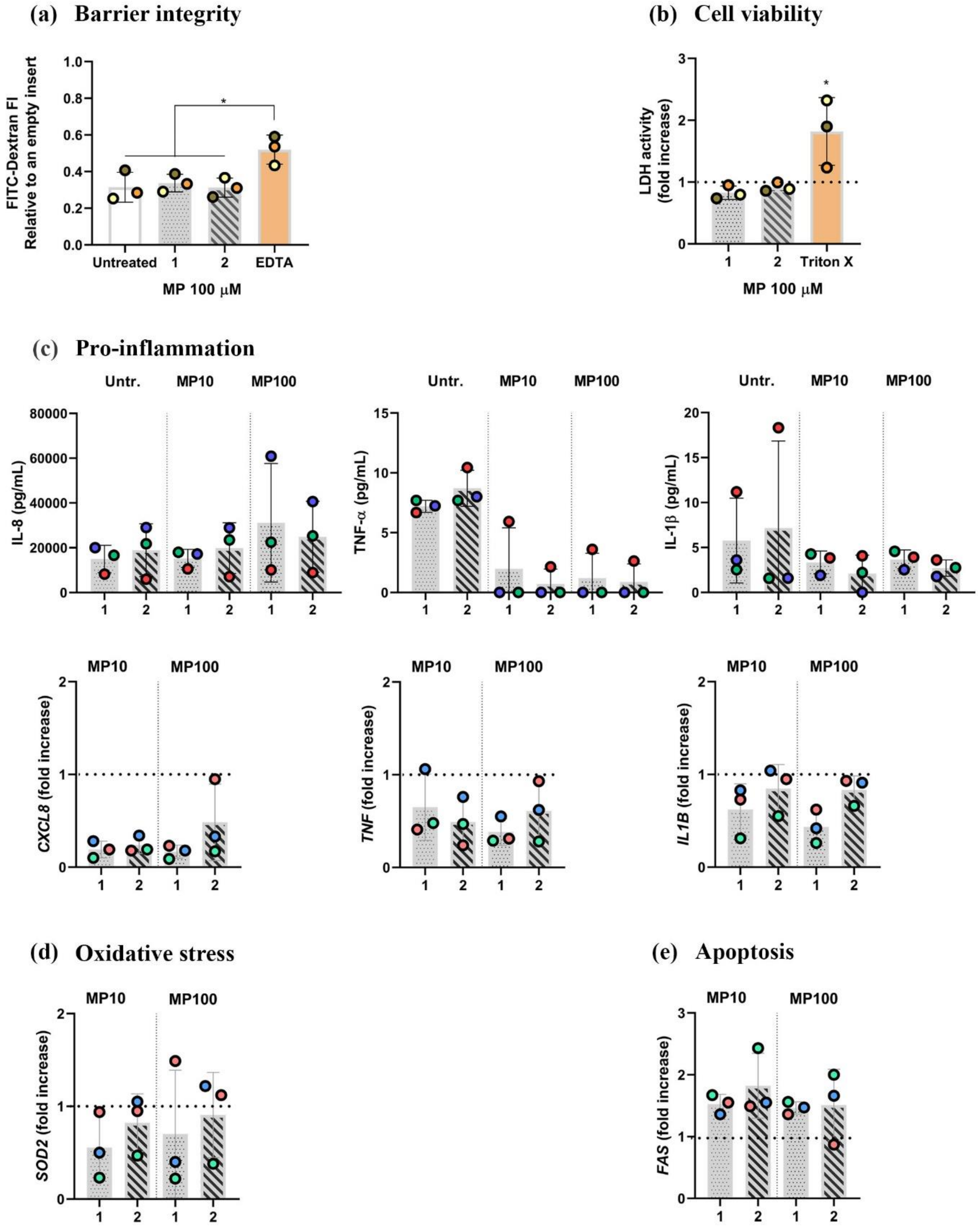

Figure S4: Absence of MP effect on healthy tissue samples upon exposure to MP for 24 h from either apical (Approach 1) or basal compartment (approach 2). (a) Cell viability assessed via a membrane rupture assay based on the LDH enzyme released in the cell culture medium of the basal compartment, presented as fold increase over untreated cells (dotted line). Positive control cells were apically exposed to Triton X-100 (0.2%; 24 h). (b) Barrier integrity, assessed via tissue permeability to FITC-Dextran (70 kDa). Data is presented as fluorescence intensity of FITC-Dextran 70 kDa, measured in the basal compartment, normalized to the values for empty inserts, as the mean of three biological repetitions (height of the column) ± standard deviation. In contrast, individual values from biological repetitions are presented as circles, color-coded according to data from the same biological repetitions. EDTA (5 mM, 60 min) was used as a positive control for barrier disruption. Statistically significant differences to untreated cells (LDH assay) or among the groups (one-way ANOVA, Tukey's post hoc; α = 0.05): *

$p \leq 0.05$. (c) Pro-inflammatory cytokine secretion and gene expression, assessed via real-time RT-qPCR. (d) oxidative stress, and (e) apoptotic gene expression. The data is shown as fold increase of mRNA calculated via the ΔΔCt method, i.e., normalized to the expression of the housekeeping gene GAPDH and to the expression of the gene of interest in the untreated samples. Dotted lines denote the mean value of untreated cells. Abbreviations: MP10 and MP100: models treated with LPS (24 h) followed by methylprednisolone at 10 or 100 μM for an additional 24 h; LPS: models exposed to LPS for 48 h from either apical (approaches 1) or basal (approach 2; 200 μL at the pseudo-air-liquid interface) compartments. Genes: *CXCL8*: interleukin 8; *TNF*: tumor necrosis factor α; *IL1B*: interleukin 1β; *SOD2*: superoxide dismutase; *FAS*: Fas cell surface death receptor.
